# FLS2‐RBOHD module regulates changes in the metabolome of *Arabidopsis* in response to abiotic stress

**DOI:** 10.1002/pei3.10101

**Published:** 2023-02-09

**Authors:** Xiaole Yu, Zhixin Liu, Aizhi Qin, Yaping Zhou, Zihao Zhao, Jincheng Yang, Mengke Hu, Hao Liu, Yumeng Liu, Susu Sun, Yixin Zhang, Masood Jan, George Bawa, Xuwu Sun

**Affiliations:** ^1^ State Key Laboratory of Cotton Biology, State Key Laboratory of Crop Stress Adaptation and Improvement, Key Laboratory of Plant Stress Biology, School of Life Sciences Henan University Kaifeng China

**Keywords:** drought, FLS2, metabolome, RBOHD, salt, stress, transcriptome

## Abstract

Through crosstalk, FLAGELLIN SENSITIVE 2 (FLS2) and RESPIRATORY BURST OXIDASE HOMOLOG D (RBOHD) are involved in regulating the homeostasis of cellular reactive oxygen species (ROS) and are linked to the metabolic response of plants toward both biotic and abiotic stress. In the present study, we examined the metabolome of *Arabidopsis* seedlings under drought and salt conditions to better understand the potential role of FLS2 and RBOHD‐dependent signaling in the regulation of abiotic stress response. We identified common metabolites and genes that are regulated by FLS2 and RBOHD, and are involved in the response to drought and salt stress. Under drought conditions, D‐aspartic acid and the expression of associated genes, such as *ASPARAGINE SYNTHASE 2* (*ASN2*), increased in both *fls2* and *robed/f* double mutants. The accumulation of amino acids, carbohydrates, and hormones, such as L‐proline, D‐ribose, and indoleacetaldehyde increased in both *fls2* and *rbohd/f* double mutants under salt conditions, as did the expression of related genes, such as *PROLINE IMINOPEPTIDASE*, *PHOSPHORIBOSYL PYROPHOSPHATE SYNTHASE 5*, and *NITRILASE 3*. Collectively, these results indicate that the FLS2‐RBOHD module regulates plant response to drought and salt stress through ROS signaling by adjusting the accumulation of metabolites and expression of genes related to metabolite synthesis.

## Graphical abstract

To explore the potential signaling pathway that is required for the quick induction of stress‐related metabolites in plants in response to drought and salt stress, in this study, we studied the changes of differentially abundant metabolites in the *fls2* mutant and the *rbohd rbohf* (*rbohd/f*) double mutant and revealed a new metabolomic perspective on the role of FLAGELLIN SENSITIVE 2 and RESPIRATORY BURST OXIDASE HOMOLOG D in regulating metabolites accumulation in response to drought and salt stress conditions

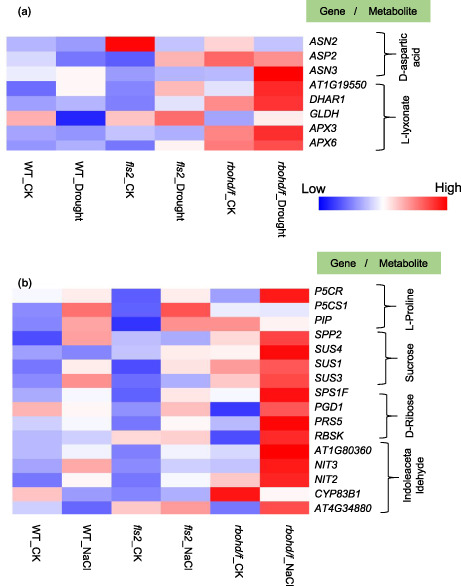

## 1 INTRODUCTION

During growth and development, plants continuously interact with the environment during their growth and development, inducing the synthesis of many different primary and secondary metabolites (Yang et al., 2018). Different metabolites are specifically or non‐specifically expressed in different developmental stages and tissues of plants, as well as in response to a variety of biotic and abiotic stresses (Isah, 2019). In this regard, drought and salt stress can have a significant negative impact on plant growth and development, and also induce dynamic changes in the level of different metabolites (Arif et al., 2020; Kumar et al., 2021). This includes metabolites such as amino acids, carbohydrates, and also secondary metabolites, such as phenolic acids and flavonoids, terpenes and steroids, and alkaloids (Isah, 2019; Singh et al., 2020). Notably, studies have indicated that these metabolites play a key role in plant adaptation to biotic and abiotic stresses (Fàbregas & Fernie, 2019). A recent study reported a higher accumulation of L‐aspartic acid in leaves of tolerant varieties of chickpea (*Cicer arietinum* L.) under drought stress conditions, indicating that L‐aspartic acid may serve as a marker metabolite for drought response (Khan et al., 2019). The concentration of soluble protein and proline, as well as superoxide dismutase and catalase activity, was also found to be elevated in Apiaceae (*Bupleurum chinense* DC.) in response to drought stress (Yang et al., 2020). Collectively, studies have shown that the synthesis of a series of metabolites is induced as part of plant response to adverse conditions.

FLAGELLIN SENSITIVE 2 (FLS2) and RESPIRATORY BURST OXIDASE HOMOLOG D (RBOHD) function in regulating cytosolic calcium and the production of reactive oxygen species (ROS) during the defense response in plants (Chi et al., 2021; Li et al., 2014; Melotto et al., 2006). Receptor‐like cytoplasmic kinase BIK1 (BOTRYTIS‐INDUCED KINASE 1), one of the components of the FLS2 immunoreceptor complex, positively regulates the accumulation of ROS by directly phosphorylating RBOHD (Li et al., 2014). FLS2 forms a functional complex with the BRASSINOSTEROID INSENSITIVE 1 (BRI1)‐associated kinase receptor 1 (BAK1), resulting in Ca^2+^ influx and ROS production (Chinchilla et al., 2007). Abiotic stresses, such as salt stress, can also enhance the accumulation of ROS in plants by activating RBOHD (Luo et al., 2021). ROS accumulation and the activation of the Ca^2+^ signaling pathway are known to be involved in abiotic stress response in plants (Bush, 1995; Yu et al., 2002). Notably, our recent study revealed the essential roles of the FLS2‐RBOHD‐PIF4 module in regulating the adaptive response of plants to drought and salt stress (Liu et al., 2022). Although FLS2‐RBOHD module has been studied on how plants respond to abiotic stress, the effects of FLS2‐RBOHD model on metabolite synthesis and level changes in plants under abiotic stress conditions have been less studied.

Therefore, we used liquid chromatograph‐mass spectrometry (LC–MS) to analyze the dynamic changes in metabolite levels in an *fls2* mutant and an *rbohd rbohf* (*rbohd/f*) double mutant under drought and salt stress conditions. The transcriptomes of the *fls2* mutant and *rbohd/f* double mutant under drought and salt stress conditions were also characterized. Differentially abundant metabolites (DAMs) in the *fls2* mutant and *rbohd/f* double mutant were identified and these data were combined with the transcriptome data and analyzed. Key metabolites and their corresponding regulatory genes that may be involved in regulating plant response to drought and salt stress conditions were identified. Our results provide novel insight that contributes to a comprehensive understanding of the mechanisms by which FLS2 and RBOHD regulate plant response to abiotic stress through ROS signaling by adjusting the accumulation of metabolites.

## 2 EXPERIMENTAL PROCEDURES

### 2.1 Plant material and growth conditions

The WT is Columbia (Col‐0). The *fls2* (SALK_141277) and *rbohd/f* double mutants (CS9558) in Col‐0 background were obtained from the *Arabidopsis* Biological Resource Center (Table S1). Homozygous T‐DNA insertion lines were confirmed by polymerase chain reaction (PCR) using gene‐specific and T‐DNA‐specific primers (Table S2; Figure S3). For NaCl treatments, 1‐week‐old seedlings were first transplanted into the soil to grow for 1 week under normal growth conditions. Subsequently, these were watered with an aqueous solution containing 100 mM NaCl and allowed to grow further for 1 week. For drought treatment, the seedlings were first transplanted into normal watered soil; afterward, watering was stopped after transplantation. After 1 week, the soil water content decreased to about 10%, and the seedlings were further grown for 1 week. For control, seedlings of the same batch were transplanted into the soil and grown under normal watering conditions (watering once a week) for 3 weeks.

### 2.2 RNA‐seq analysis

Total RNA was extracted using the mirVana miRNA isolation kit (Ambion) following the manufacturer's protocol. RNA integrity was evaluated using an Agilent 2100 Bioanalyzer (Agilent Technologies). The samples with RNA Integrity Number (RIN) ≥ 7 were subjected to subsequent RNA‐seq analysis. The libraries were constructed using TruSeq Stranded mRNA LTSample Prep Kit (Illumina) according to the manufacturer's instructions. Libraries were sequenced on the Illumina sequencing platform (HiSeqTM 2500 or Illumina HiSeq X Ten), and 125‐bp/150‐bp paired‐end reads were generated. Then, raw data (raw reads) were processed using Trimmomatic. The reads containing ploy‐N and the low‐quality reads were removed to obtain clean reads. Then, the clean reads were mapped to the reference genome using hisat2. After that, the FPKM value of each gene was calculated using cufflinks, and the read counts of each gene were obtained by htseq‐count. DEGs were identified using the DESeq (2012) R package functions estimateSizeFactors and nbinomTest. *p*‐value <.05 and fold change >2 or fold change <.5 were set as the threshold for significantly differential expression. Hierarchical cluster analysis of DEGs was performed to explore gene expression patterns. RNA sequence data are available at https://dataview.ncbi.nlm.nih.gov/?search=SUB8234436 (Liu et al., 2022).

### 2.3 LC–MS analysis

All chemicals and solvents were analytical or HPLC grade. Water, methanol, acetonitrile, and formic acid were purchased from CNW Technologies GmbH. L‐2‐chlorophenylalanine was purchased from Shanghai Hengchuang Bio‐technology Co., Ltd.

### 2.4 Sample preparation

Transfer 80 mg of accurately weighed sample to a 1.5 mL Eppendorf tube. Two small steel balls were added to the tube. 20 μL of 2‐chloro‐l‐phenylalanine (0.3 mg/mL) dissolved in methanol as internal standard and a 1 mL mixture of methanol and water (7/3, vol/vol) were added to each sample, samples were placed at −80°C for 2 min. Then grinded at 60 HZ for 2 min, and ultrasonicated at ambient temperature for 30 min after vortexed, then placed at 4°C for 10 min. Samples were centrifuged at 13,000 rpm, 4°C for 15 min. Supernatant in a brown and glass vial was dried in a freeze concentration centrifugal dryer. A mixture of methanol and water (1/4, vol/vol) were added to each sample, samples vortexed for 30 s, then placed at 4°C for 2 min. Samples were centrifuged at 13,000 rpm, 4°C for 5 min. The supernatants (150 μL) from each tube were collected using crystal syringes, filtered through 0.22 μm microfilters, and transferred to LC vials. The vials were stored at −80°C until LC–MS analysis. QC samples were prepared by mixing aliquots of all samples to be a pooled sample.

### 2.5 Sample on‐board processing

An ACQUITY UHPLC system (Waters Corporation) coupled with an AB SCIEX Triple TOF 5600 System (AB SCIEX) was used to analyze the metabolic profiling in both ESI‐positive and ESI‐negative ion modes. An ACQUITY UPLC BEH C18 column (1.7 μm, 2.1 × 100 mm) was employed in both positive and negative modes. The binary gradient elution system consisted of (A) water (containing 0.1% formic acid, v/v) and (B) acetonitrile (containing 0.1% formic acid, v/v) and separation was achieved using the following gradient: 0 min, 5% B; 2 min, 20% B; 4 min, 25% B; 9 min, 60% B; 14 min, 100% B; 18 min, 100% B; 18.1 min, 5% B and 19.5 min, 5% B The flow rate was 0.4 mL/min and column temperature was 45°C. All the samples were kept at 4°C during the analysis. The injection volume was 2 μL. Data acquisition was performed in full scan mode (m/z ranges from 70 to 1000) combined with IDA mode. The QCs were injected at regular intervals (every 10 samples) throughout the analytical run to provide a set of data from which repeatability can be assessed. Mass spectrum conditions: ESI was used as ion source. Positive and negative ion scanning modes were used to collect the sample quality spectrum signals. The mass spectrum parameters are shown in Table 1.

**TABLE 1:** Mass spectrum parameter

Parameter	Positive ion	Negative ion
Spray voltage (V)	3800	3200
Capillary temperature (°C)	320	320
Aux gas heater temperature (°C)	350	350
Sheath gas flow rate (Arb)	35	30
Aux gas flow rate (Arb)	8	0
S‐lens radio frequency level	50	50
Mass range (m/z)	100–1000	100–1000
Full ms resolution	70,000	70,000
MS (Mass)/MS resolution	17,500	17,500
NCE (Normalized Collisional Energy)/stepped NCE	10, 20, 40	10, 20, 40

### 2.6 LC–MS data preprocessing and statistical analysis

The acquired LC–MS raw data were analyzed by the progenesis QI software (Waters Corporation) using the following parameters. The resulting matrix was further reduced by removing any peaks with a missing value (ion intensity = 0) in more than 50% samples. The internal standard was used for data QC (quality control) (reproducibility). Metabolites were identified by progenesis QI (Waters Corporation) Data Processing Software, based on public databases such as http://www.hmdb.ca/; http://www.lipidmaps.org/ and self‐built databases. The positive and negative data were combined to get a combined data which were imported into R ropls package. Principal component analysis (PCA) and (orthogonal) partial least‐squares‐discriminant analysis (O) PLS‐DA were carried out to visualize the metabolic alterations among experimental groups, after mean centering (Ctr) and Pareto variance (Par) scaling, respectively. PCA is to explore the degree of correlation among multiple possible correlation variables, find the maximum or minimum correlation direction, and achieve the purpose of data compression or noise reduction (dimension reduction). OPLS‐DA analysis combines orthogonal signal correction and PLS‐DA methods to decompose the X matrix into two types of information related and unrelated to Y, and screen the differential variables by removing the unrelated differences. The abscissa and ordinate of the PCA graph represent the projected score values of each sample on the PC1 (principal components 1) and PC2, respectively (Nicholson et al., 1999). The projected score value of each sample on the plane is composed of the first principal component and the second principal component is the spatial coordinate, which can intuitively reflect the similarity or difference between the samples (Okada et al., 2010). The closer the distance between different samples indicates the closer the composition and concentration of the molecules they contain (Jolliffe & Cadima, 2016). On the OPLS‐DA graph, there are two principal components, the predicted PC1 and the orthogonal principal component (PCo1) (Trygg & Wold, 2002). OPLS‐DA maximizes the differences between groups and reflects the PC1, so the differences between groups can be directly distinguished from the PC1, while the PCo1 reflects the intra‐group differences (Trygg & Wold, 2002). The Hotelling's T2 region, shown as an ellipse in score plots of the models, defines the 95% confidence interval of the modeled variation. Variable importance in the projection (VIP) ranks the overall contribution of each variable to the OPLS‐DA model, and those variables with VIP >1 are considered relevant for group discrimination. In this study, the default 7‐round cross‐validation was applied with one‐seventh of the samples being excluded from the mathematical model in each round, in order to guard against overfitting. The differential metabolites were selected based on the combination of a statistically significant threshold of VIP values obtained from the OPLS‐DA model and p values from a two‐tailed Student's *t*‐test on the normalized peak areas, where metabolites with VIP values larger than 1.0 and *p* values less than .05 were considered as differential metabolites (Tables S3–S24). Metabolites involved in metabolic pathway analysis using MetaboAnalyst5.0: https://www.metaboanalyst.ca/ (Pang et al., 2022).

### 2.7 Association analysis of metabolome and transcriptome

Metabolite synthesis‐related genes were found according to the metabolic pathways involved in metabolites in the KEGG: https://www.kegg.jp/. The genes involved in metabolite‐related metabolic pathways were compared with the transcriptome data obtained by RNA‐seq to obtain the expression levels of genes in different samples. The heat map of gene expression was drawn using the website of Oebiotech: https://cloud.oebiotech.com/task/detail/heatmap/. And the GO analyses were performed on Metascape: http://metascape.org (Zhou et al., 2019). Firstly, the metabolite synthesis‐related genes list was uploaded on Metascape according to the operating manual. After uploading the data, GO analyses were performed on Metascape automatically. After the analysis was completed, the results of GO were downloaded from Metascape.

## 3 RESULTS

### 3.1 LC–MS analysis of *fls2* mutant and *rbohd/f* double mutant under no‐stress (CK), salt, and drought stress conditions

We previously demonstrated that an *Arabidopsis rbohd/f* double mutant exhibited sensitivity to drought and salt stress, relative to wild‐type (WT) and *fls2* mutant plants which exhibited tolerance to both stresses (Liu et al., 2022). Therefore, we analyzed the metabolome of WT, *fls2* mutant, and *rbohd/f* double mutant under drought and salt stress conditions to determine if FLS2 and RBOHD/F regulate the response of plants to drought and salt stress through their ability to mediate the accumulation of specific metabolites. An LC–MS full scan was first used to obtain data on the ionic strength of different molecules (Figure S1a). We determined the intensity of the strongest ions at each retention time point in *fls2*, *rbohd/f*, and WT plants under salt stress, drought stress, and CK conditions (Figure S1a). Differences in the number of peaks and ion intensity in each of the graphs derived from the different samples indicate that the types and amounts of metabolites differed in each sample under the different treatment conditions (Figure S1a).

A PCA diagram illustrates that the samples clustered together, and therefore indicates that there was little difference between samples (Figure S2). The metabolomic data obtained in the experiment were multidimensional and some of the variables were highly correlated. Therefore, we utilized a multivariate statistical analysis to identify DAMs between different comparison groups.

Principal component analysis was first used to observe the overall distribution of DAMs in different samples (Figure S1b). Then, orthogonal projections to latent structures discriminant analysis (OPLS‐DA) was used to determine differences between samples (Figure S1c). DAMs with biological significance were thus identified through the OPLS‐DA analysis. In addition, we also used fold change to further confirm the significance of the DAMs between comparison groups (Figures 2a, 4a, and 6a; Figure S5a).

### 3.2 FLS2 is involved in sensing salt stress

FLAGELLIN SENSITIVE 2 is involved in sensing the bacterial flagellin *flg22*, which in turn leads to a transient increase in cytosolic calcium ions and ROS (Chinchilla et al., 2006; Gómez‐Gómez & Boller, 2000). In the present study, we characterized changes in the metabolome and transcriptome of *fls2* mutant and WT plants under CK and NaCl conditions to determine the potential involvement of FLS2‐mediated ROS signaling in response to salt stress.

**FIGURE 1:**
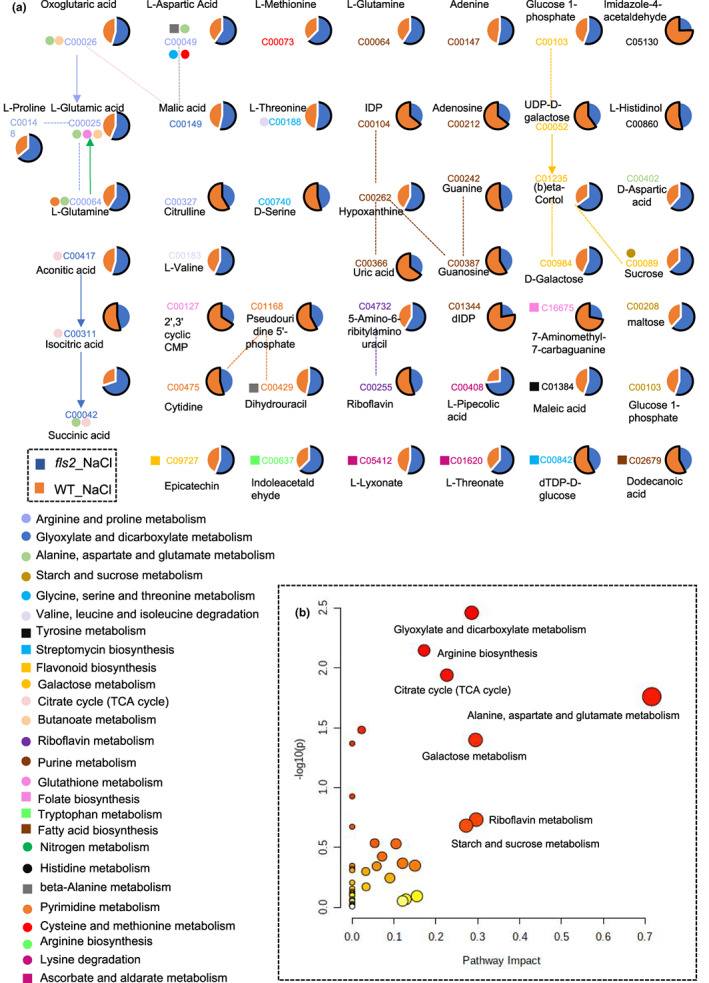
Metabolic network diagram of differentially abundant metabolites (DAMs) in *fls2*_NaCl and WT_NaCl samples. (a) Metabolic network diagram of DAMs in the comparison between *fls2*_NaCl and WT_NaCl samples. Solid lines indicate one‐step reactions; dashed lines indicate two‐ or more‐step reactions. (b) Impact of DAMs in *fls2*_NaCl versus WT_NaCl comparisons on different metabolic pathways. All matching pathways for metabolites are shown based on *p*‐values from pathway enrichment analysis and pathway impact values are derived from pathway topology analysis. The color of the circle is based on the *p*‐values, from light to dark, and the *p*‐value from large to small. The radius of the circle is based on its pathway impact value, from small to large, and the impact value from small to large. The pathway impact value is the ratio of the sum of the importance measures of each matched metabolite to the importance measures of all metabolites in each pathway.

Principal component analysis indicated that the distance between *fls2* mutant and WT plants under salt stress conditions is relatively far, suggesting that the types and abundance of metabolites in *fls2* mutant plants were different from WT plants under salt stress conditions (Figure S1b). We then identified the DAMs in *fls2* mutant samples and WT samples under CK and NaCl conditions (VIP >1, *p* < .05) via LC–MS analysis. The metabolic pathway matching analysis of the identified DAMs was conducted utilizing the exact mass number, MetaboAnalyst (www.metaboanalyst.ca/), and Kyoto Encyclopedia of Genes and Genomes (KEGG) (Figure 1a,b). We focused on metabolites that were annotated in the KEGG database, especially those known to be development‐related metabolites and pathways (Figure 1a,b). Results identified 48 DAMs in the comparison between “*fls2*_NaCl versus WT_NaCl” that were assigned to 26 metabolic pathways mainly associated with plant development, including: “arginine biosynthesis,” “galactose metabolism,” and “citrate cycle (TCA cycle)” (Figure 1a,b).

**FIGURE 2:**
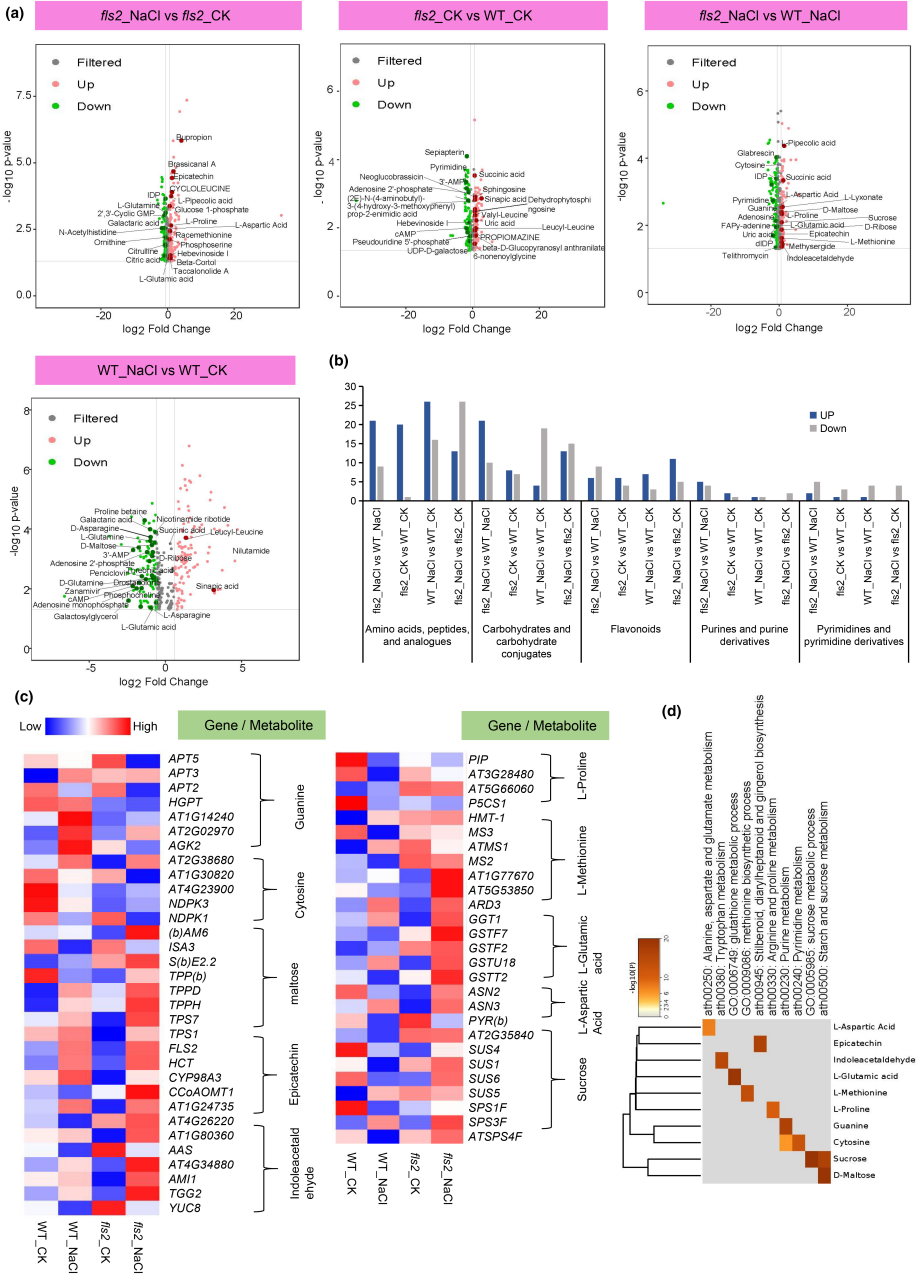
Identification and analysis of the differentially abundant metabolites (DAMs) in the comparison between “*fls2*_NaCl versus WT_NaCl” samples. (a) Volcano plots of DAMs were identified in the various comparisons of *fls2* and WT under CK and salt stress conditions. DAMs were identified based on a *p*‐value <.05 and a |log2FC| > .58. Red dots indicate significantly higher abundant metabolites in the comparison groups, green dots indicate significantly lower abundant metabolites, and gray dots indicate metabolites with insignificant changes in abundance. (b) Bar chart of the number of metabolites in different categories of metabolites in “*fls2*_NaCl versus WT_NaCl” samples. The height of the blue bars represents the number of metabolites with increased abundance in the different comparison groups. The gray bars represent the number of metabolites with decreased abundance. (c) Heat map of metabolite‐related gene expression in “*fls2*_NaCl versus WT_NaCl” comparisons. The heat map indicates the expression of genes related to L‐aspartic acid, L‐proline, L‐glutamic acid, L‐methionine and sucrose, maltose, epicatechin, indoleacetaldehyde, guanine, and cytosine in *fls2*_NaCl, *fls2*_CK, WT_CK, WT_NaCl samples. (d) Heat map of gene expression of metabolite synthesis‐related genes identified in GO enrichment analysis. Genes presented are related to (c).

Next, we analyzed changes in the abundance of these metabolites. Results indicated that the number of amino acids and carbohydrates with increased abundance were greater in *fls2*_NaCl than in WT_NaCl, including L‐proline, L‐glutamic acid, and maltose (Figure 2a,b). Under normal growth conditions in the comparison group of “*fls2*_ CK versus WT_CK,” the number of flavonoids with increased expression in *fls2* mutant is higher than WT. In contrast, under NaCl treatment conditions, in the comparison group of “*fls2*_Nacl versus WT_NaCl,” the number of flavonoids with increased expression in *fls2* mutant is lower than WT (Figure 2b). The high abundance of the identified metabolites in the *fls2* mutant indicates that the *fls2* mutant was sensitive to salt stress. Notably, we also found that the abundance of indole acetaldehyde was significantly higher in the “*fls2*_NaCl versus WT_NaCl” comparison.

An association analysis of the transcriptome and metabolome data was then performed to determine if changes in the expression of genes related to the DAMs were following each other. As shown in Figure 2c, we focused on DAMs such as L‐aspartic acid, L‐proline, L‐glutamic acid, L‐methionine, sucrose, maltose, epicatechin, indoleacetaldehyde, guanine, and cytosine in the correlation analysis (Figure 2c). The expression data (transcriptome) of metabolite‐related genes were used to construct a heatmap and GO process chart (Figure 2c,d). The results indicated that in “*fls2*_NaCl versus WT_NaCl,” “*fls2*_CK versus WT_CK,” “*fls2*_NaCl versus *fls2*_CK,” and “WT_NaCl versus WT_CK” comparisons, changes in the expression of metabolite‐related genes exhibited similar trends to changes in the abundance of metabolites. For example, the expression level of the L‐glutamic acid‐related genes *gamma‐glutamyl transpeptidase 1* (*GGT1*) and *glutathione s‐transferase tau 18* (*GSTU18*) were upregulated in the “WT_NaCl versus WT_CK” and “*fls2*_NaCl versus WT_NaCl” (log_2_FC >0) comparison groups, which is consistent with the increase of L‐glutamic acid in “*fls2*_NaCl versus WT_NaCl” comparison group (Figure 2a,c). Additionally, the guanine‐related gene *GUANYLATE KINASE*—(*AGK2* or *GK‐2*) was downregulated in “*fls2*_NaCl versus WT_NaCl” and “*fls2*_NaCl versus *fls2*_CK” comparison groups, which is consistent with the changes of observed in guanine abundance (Figure 2a,c). Furthermore, as shown in Figure 2c, we also found that genes related to metabolites such as sucrose and indole acetaldehyde were significantly upregulated in the “*fls2_*NaCl versus *fls2_*CK” comparison group (Figure 2c), indicating that the *fls2* mutant is either highly sensitive to salt stress or that FLS2 is involved in sensing salt stress signals.

### 3.3 Increased salt stress sensitivity of *rbohd/f* double mutant plants

Excessive soil salinity induces an increase in ROS that is regulated by RBOHD/F (Xie et al., 2011). To protect cells from the harmful effects of excessive levels of ROS produced under salt stress conditions, plants have developed a variety of antioxidant defense mechanisms to maintain the homeostasis of the intracellular redox state (Acosta‐Motos et al., 2017). Therefore, we conducted an analysis of the metabolome and transcriptome of the *rbohd/f* double mutant and WT plants under normal and salt stress conditions to examine the effect of the ROS signal generated by RBOHD/F on the growth of plants under salt stress conditions.

**FIGURE 3:**
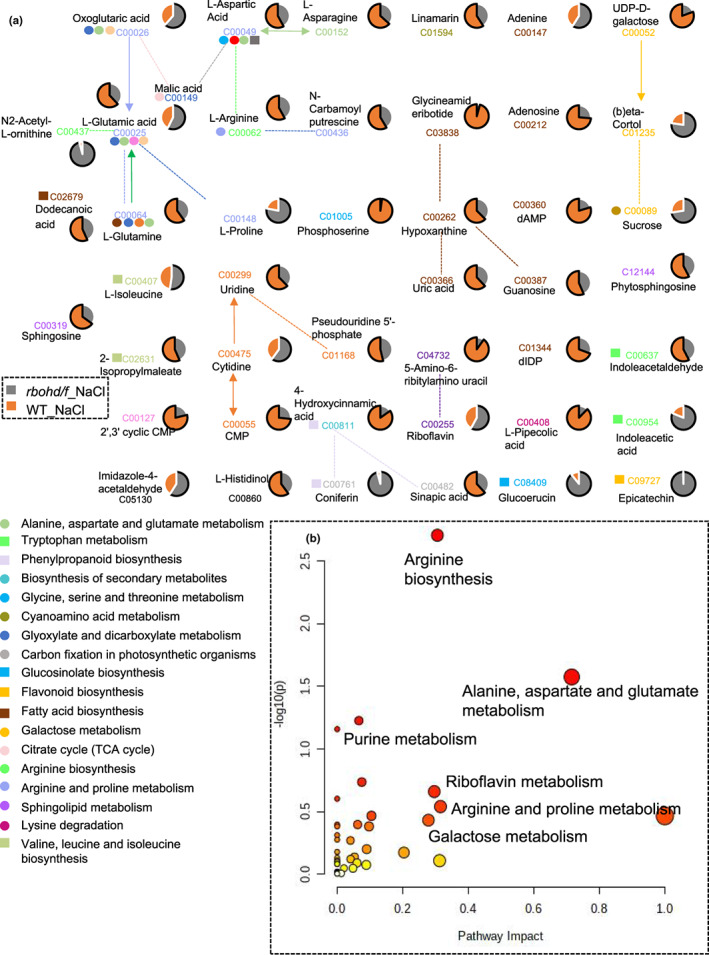
Metabolic network diagram of differentially abundant metabolites (DAMs) in the “*rbohd/f*_NaCl versus WT_NaCl” comparison. (a) Metabolic network diagram of DAMs in the “*rbohd/f_*NaCl versus WT_NaCl” comparison group. Solid lines indicate one‐step reactions; dashed lines indicate two‐ or more‐step reactions. (b) Impact of DAMs in the “*rbohd/f*_NaCl versus WT_NaCl” comparison group on different metabolic pathways. Matching pathways for metabolites were selected based on *p*‐values in a pathway enrichment analysis and pathway impact values were derived from pathway topology analysis.

Principal component analysis indicated that the distance between *rbohd/f* double mutant and WT samples was considerable in the “*rbohd/f*_NaCl versus WT_NaCl” comparison, suggesting that the level of metabolites in *rbohd/f* plants was altered in response to salt stress, relative to WT plants (Figure S1b). A total of 45 DAMs were identified in the “*rbohd/f*_NaCl versus WT_NaCl” comparison group that were assigned to 18 metabolic pathways, including “arginine and proline metabolism,” “alanine, aspartate and glutamate metabolism,” and “riboflavin metabolism” (Figure 3a,b).

**FIGURE 4:**
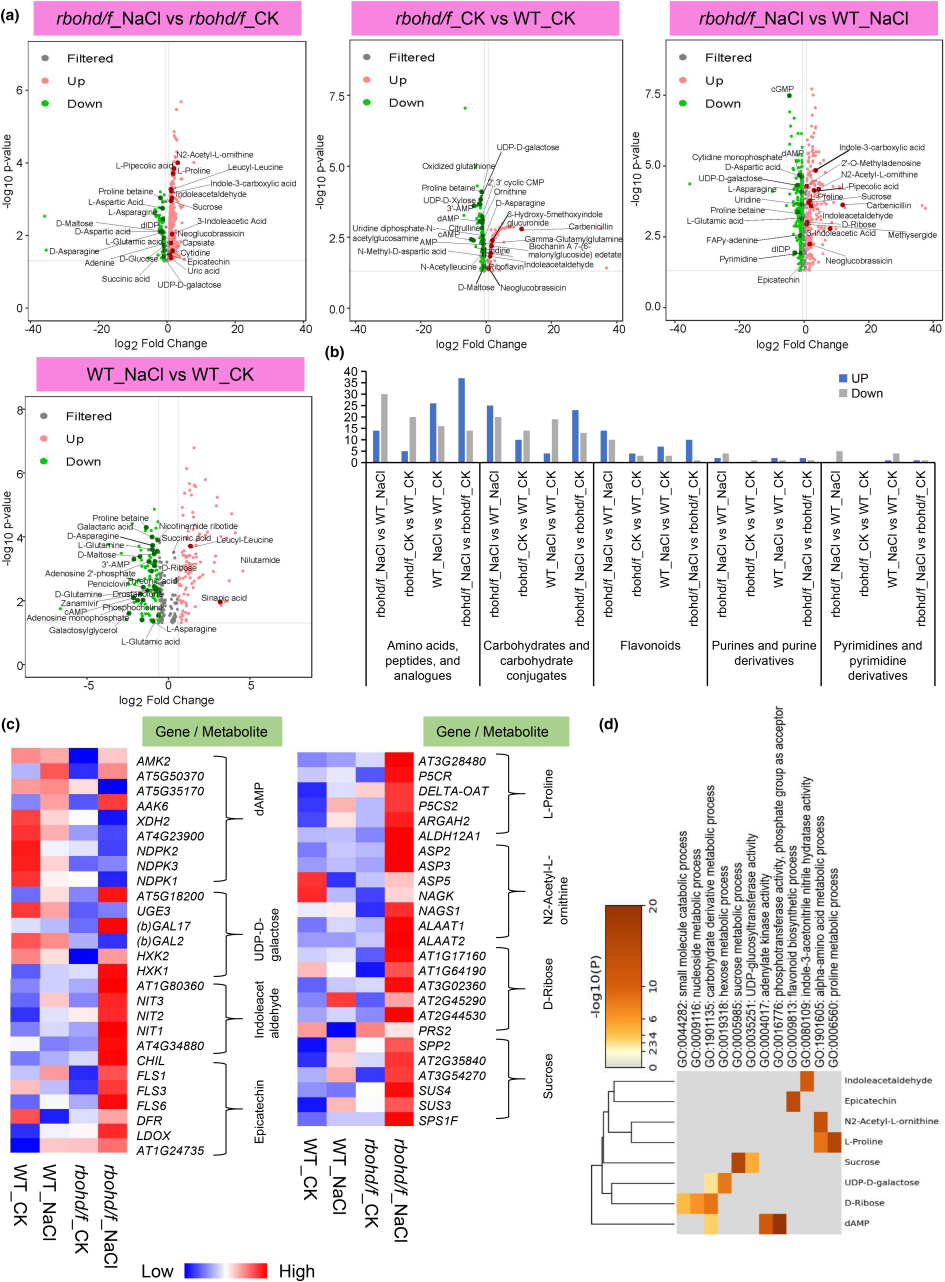
Identification and analysis of the differentially abundant metabolites (DAMs) in different comparison groups under CK and salt stress conditions. (a) Volcano plots of DAMs were identified in the various comparisons of *rbohd/f* and WT samples under CK and salt stress conditions. DAMs were determined based on *p*‐value and fold change (*p*‐value < .05, |log2FC| > .58). Red dots indicate metabolites with significantly increased abundance, green dots represent metabolites with significantly decreased abundance, and gray dots indicate a change in metabolites that were nonsignificant. (b) Bar chart of the number of metabolites in different categories of metabolites in the “*rbohd/f*_NaCl versus WT_NaCl,” “*rbohd/f*_CK versus WT_CK,” “WT_NaCl versus WT_CK,” and “*rbohd/f*_NaCl versus *rbohd/f* _CK” comparison groups. The height of the blue bars represents the number of metabolites with increased abundance in the different comparison groups. The gray bars represent the number of metabolites with decreased abundance. (c) Heat map of metabolite‐related gene expression in the different *rbohd/f*_NaCl versus WT_NaCl comparisons. Heat map of metabolite‐related gene expression in *rbohd/f*_NaCl, *rbohd/f*_CK, WT_CK, and WT_NaCl samples. Heat map indicates gene expression levels for genes related to L‐proline, N2‐acetyl‐L‐ornithine, D‐ribose, sucrose, indoleacetaldehyde, epicatechin, dAMP, and UDP‐D‐galactose. (d) Heat map of gene expression of metabolite synthesis‐related genes identified in GO enrichment analysis. Genes presented are related to (c).

The number of amino acids, carbohydrates, and flavonoids with increased abundance was greater in *rbohd/f*_NaCl samples, relative to *rbohd/f*_CK samples, including metabolites such as N2‐acetyl‐L‐ornithine, sucrose, and epicatechin. The number of amino acids, carbohydrates, and flavonoids with increased abundance in the “*rbohd/f*_NaCl versus *rbohd/f*_CK” comparison group also increased, relative to the “WT_NaCl versus WT_CK” comparison group (Figures 3a and 4a,b). Moreover, *rbohd/f*_NaCl samples exhibited an increase in the numbers of carbohydrates and flavonoids, compared to WT_NaCl samples, as was well as an increase in the abundance of auxin‐related metabolites such as indole acetaldehyde and indoleacetic acid (Figure 4a,b). The number of amino acids with increased abundance also increased in the “*rbohd/f*_NaCl versus WT_NaCl” comparison group, relative to the “*rbohd/f*_CK versus WT_CK” comparison group (Figure 4b). Most of the metabolites with increased abundance were involved in “galactose metabolism” and “arginine and proline metabolism,” which contribute to the energy supply available for plant growth, as well as plant defense (Abedi et al., 2018; Majumdar et al., 2016) (Figure 3a,b). In contrast, the number of pyrimidine and purine metabolites decreased in *rbohd/f*_NaCl samples in the “*rbohd/f*_NaCl versus WT_NaCl” comparison group, including cGMP and UDP‐D‐galactose (Figure 4b). We also found that the number of carbohydrate metabolites that decreased in abundance was higher than the number of carbohydrate metabolites with increased abundance in the “*rbohd/f*_CK versus WT_CK” comparison group (Figure 4b).

Next, we performed an association analysis between the transcriptome and metabolome data obtained for the “*rbohd/f*_CK versus WT_CK,” “*rbohd/f*_NaCl versus WT_NaCl,” “WT_NaCl versus WT_CK,” and “*rbohd/f*_NaCl versus *rbohd/f* _CK” comparison groups. Association analysis focused on the expression of genes related to L‐proline, N2‐acetyl‐L‐ornithine, D‐ribose, sucrose and indoleacetaldehyde, epicatechin, dAMP, and UDP‐D‐galactose (Figure 4c,d). The analysis indicated that the expression of genes related to L‐proline, sucrose, indoleacetaldehyde, N2‐acetyl‐l‐ornithine, and D‐ribose, epicatechin synthesis in *rbohd/f*_NaCl samples was mostly higher than in other samples (Figure 4c). The expression of *N‐ACETYL‐L‐GLUTAMATE SYNTHASE 1* (*NAGS1*), *SUCROSE SYNTHASE 3* (*SUS3*) was also significantly higher in *rbohd/f*_NaCl samples than in other samples. The level of expression of genes related to dAMP and UDP‐D‐galactose in *rbohd/f*_NaCl samples, however, was lower than in WT_NaCl samples (Figure 4d). These results suggest that RBOHD/F are involved in regulating the expression of genes related to metabolites induced by salt stress.

### 3.4 Fls2 is involved in sensing drought stress signals

Plants subjected to drought stress accumulate a variety of organic and inorganic substances, including sugars, amino acids, and inorganic ions, to increase the osmotic potential of their cells and enhance their water retention capacity (Rhodes & Samaras, 1994). Therefore, we analyzed the metabolome and transcriptome of *fls2* mutant and WT plants under CK and drought conditions to examine the function of FLS2 in drought response.

**FIGURE 5:**
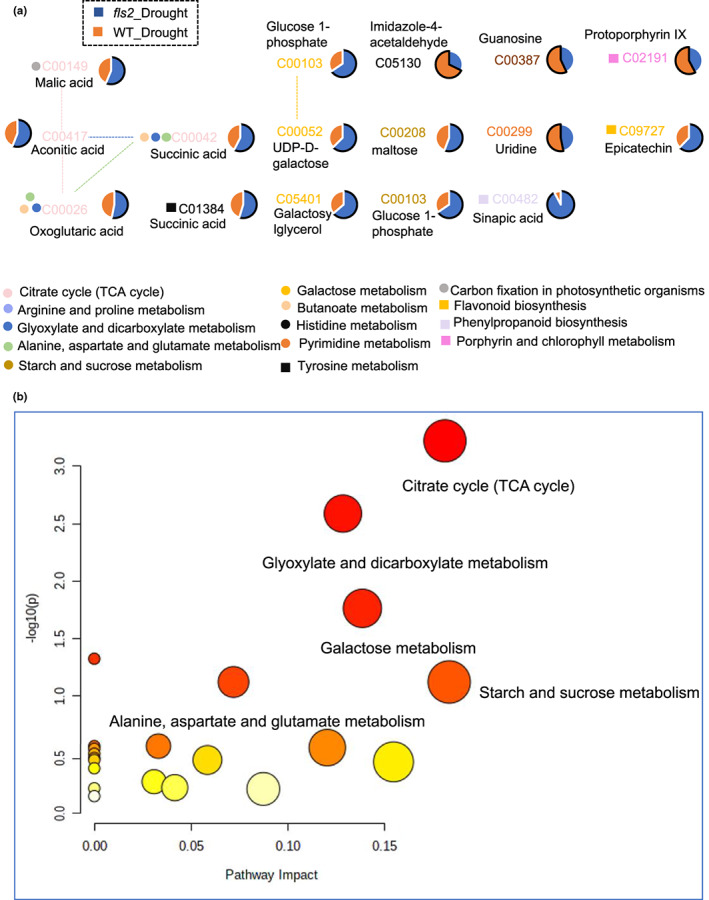
Metabolic network diagram of differentially abundant metabolites (DAMs) in the “*fls2*_Drought versus WT_Drought” comparison group. (a) Metabolic network diagrams of DAMs in the “*fls2*_Drought versus WT_Drought” comparison group. Solid lines indicate one‐step reactions; dashed lines indicate two‐or more‐step reactions. (b) The impact of DAMs in the “*fls2*_Drought versus WT_Drought” comparison on different metabolic pathways. All matching pathways for metabolites were determined based on *p*‐values obtained from a pathway enrichment analysis and pathway impact values derived from a pathway topology analysis.

Principal component analysis indicated that the *fls2* mutant subjected to drought stress did not significantly differ in their accumulation of metabolites compared to WT plants (Figure S1b). A total of 16 DAMs were identified in the “*fls2*_Drought versus WT_Drought” comparison that were assigned to 14 metabolic pathways. These included “glyoxylate and dicarboxylate metabolism,” “alanine, aspartate, and glutamate metabolism,” and the “citrate cycle (TCA cycle)” (Figure 5a,b).

**FIGURE 6:**
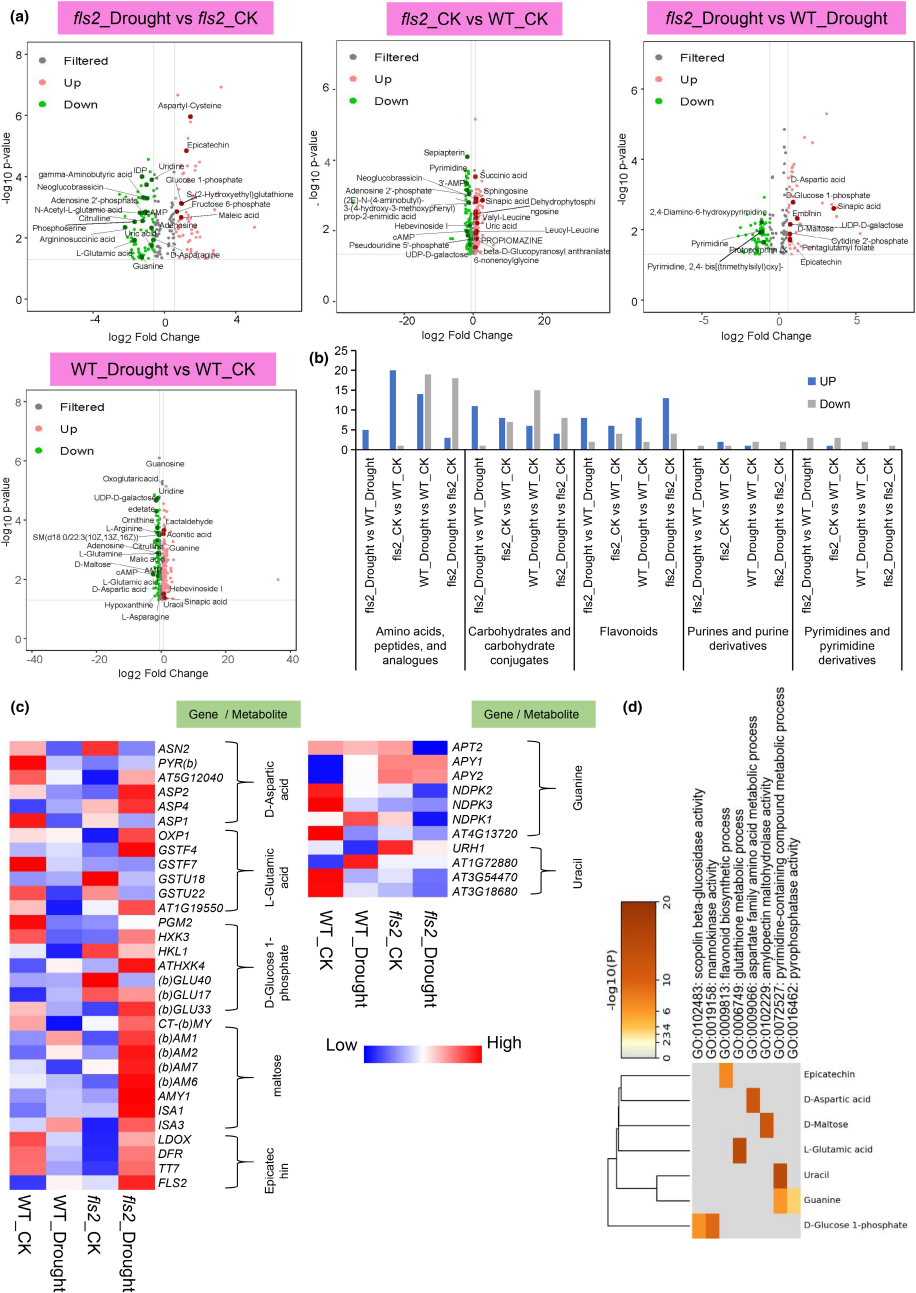
Identification and analysis of differentially abundant metabolites (DAMs) in different comparison groups of *fls2* and WT plants under CK and drought stress conditions. (a) Volcano plots of DAMs in *fls2* and WT samples under CK and drought stress conditions. DAMs were identified based on a *p*‐value < .05 and |log2FC| > .58. Red dots indicate metabolites with significantly increased abundance, green dots indicate metabolites with significantly decreased abundance, and gray dots indicate DAMs whose change in abundance was nonsignificant. (b) Bar chart of the number of metabolites in different categories of metabolites in each of the comparison groups. (c) Heat map of metabolite‐related gene expression in *fls2* and WT samples under CK and drought stress conditions. The heat map illustrates the expression level of genes related to D‐aspartic acid, L‐glutamic acid, D‐glucose 1‐phosphate, maltose, epicatechin, and guanine and uracil synthesis in *fls2*_Drought, *fls2*_CK, WT_CK, and WT_Drought samples. (d) Heat map of gene expression of metabolite synthesis‐related genes identified in GO enrichment analysis. Genes presented are related to (c).

Analysis of the “*fls2*_CK versus WT_CK” and “*fls2*_Drought versus WT_Drought” comparison groups indicated that the number of amino acids with increased abundance was lower in *fls2*_Drought plants, while the number of flavonoid metabolites and carbohydrates with higher abundance increased in the “*fls2*_Drought versus WT_Drought” comparison group (Figure 6a,b), including L‐lyxonate and epicatechin. Additionally, the number of amino acids and carbohydrates with increased abundance was lower in the “*fls2*_Drought versus *fls2*_CK” comparison group than it was in the “WT_Drought versus WT_CK” comparison group (Figure 6b). Unlike *fls2*_NaCl samples, no increase in the accumulation of indoleacetaldehyde was observed in *fls2*_Drought samples (Figures 2a and 6a).

An association analysis of metabolome and transcriptome data was performed for the DAMs of D‐aspartic acid (DAA), L‐glutamic acid, D‐glucose 1‐phosphate, maltose, epicatechin, guanine, and uracil in “*fls2*_Drought versus *fls2*_CK,” “*fls2*_CK versus WT_CK,” “*fls2*_Drought versus WT_Drought,” and “WT_Drought versus WT_CK” sample groups. Results indicated that the expression of genes related to most of the DAMs showed similar trends to their corresponding DAMs (Figure 6a,c). For example, the level of the DAA‐related gene *ASPARTATE AMINOTRANSFERASE 2* (*ASP2*) was higher in *fls2*_Drought and WT_CK samples than in *fls2*_CK and WT_Drought samples (Figure 6c). Furthermore, the expression of genes related to maltose synthesis was significantly upregulated in *fls2*_Drought samples (Figure 6c,d). The expression of genes related to epicatechin, D‐glucose 1‐phosphate, and DAA, such as *ASPARTATE AMINOTRANSFERASE 1* (*ASP1*) and *PHOSPHOGLUCOMUTASE 2* (*PGM2*), were also upregulated in WT_CK samples (Figure 6c).

### 3.5 *rbohd/f* double mutant exhibits enhanced sensitivity to drought stress

Electron transfer during photorespiration is inhibited when plants are subjected to drought stress, resulting in the production of a large amount of ROS (Noctor et al., 2014). Therefore, we performed an analysis of the metabolome and transcriptome of *rbohd/f* double mutant and WT plants under CK and drought conditions to examine the role of RBOHD/F‐mediated ROS signaling in plant response to drought stress.

A total of 37 DAMs were identified in the “*rbohd/f*_Drought versus WT_Drought” comparison group that were assigned to 23 metabolic pathways known to be involved in plant development. Most of the DAMs with increased abundance in *rbohd/f*_Drought samples are involved in “arginine biosynthesis,” “phenylpropanoid biosynthesis,” and as well as other pathways. The major metabolic pathways identified are related to response to stress and removal of ROS in plants (Amarowicz & Weidner, 2009) (Figure S4a,b).

The analysis of “*rbohd/f*_Drought versus *rbohd/f*_CK” and “WT_Drought versus WT_CK” comparison groups indicated that the number of amino acids, carbohydrates, and flavonoids with increased abundance significantly increased in *rbohd/f*_Drought samples (Figure S5b). In contrast, the number of purines and pyrimidines with increased abundance were observed to decrease in *rbohd/f*_Drought samples (Figure S5a,b). These results indicate that the abundance of a great number of metabolites in the *rbohd/f* double mutant changes under drought stress conditions. Additionally, the analysis of the “*rbohd/f*_CK versus WT_CK” and “*rbohd/f*_Drought versus WT_Drought” comparison groups indicated that the number of amino acids, flavonoids, and carbohydrate metabolites with increased abundance was significantly higher in *rbohd/f*_Drought samples (Figure S5b).

An association analysis of metabolome and transcriptome data for DAMs was conducted that include the metabolites L‐proline, L‐glutamic acid, sucrose, indoleacetic acid, AMP, and UDP‐D‐galactose. As shown in Figure S5c, results indicated that the expression of genes related to the synthesis of L‐glutamic acid, sucrose, L‐proline, and indoleacetic acid was higher in *rbohd/f* samples than in WT samples under drought stress conditions. For example, the level of the indoleacetic acid‐related genes *NITRILASE 2* (*NIT2*) and *SULFOTRANSFERASE 16* (*SOT16*) were significantly higher in *rbohd/f_*CK and *rbohd/f_*Drought samples. The level of expression of genes related to AMP and UDP‐D‐galactose synthesis was higher in WT_CK samples than in *rbohd/f_*CK samples (Figure S5a,c).

### 3.6 Expression analysis of antioxidant defense‐related genes in *fls2* and *rbohd/f* mutants

The activity and abundance of several antioxidant proteins in plants are enhanced when they are exposed to abiotic stress to provide protection from oxidative injury to cells (Gill & Tuteja, 2010). Thus, FLS2 and RBOHD/F‐mediated ROS signaling are a critical aspect of plant response to abiotic stress as demonstrated by the increased sensitivity of the *Arabidopsis rbohd/f* double mutant to abiotic stresses (Liu et al., 2022). Plants have evolved a complex enzymatic and non‐enzymatic antioxidant system to maintain the homeostasis of their intracellular redox state (Nadarajah, 2020). Protective enzymes include SUPEROXIDE DISMUTASE (SOD), CATALASE (CAT), ASCORBATE PEROXIDASE (APX), and ALTERNATIVE OXIDASE (AOX) (Mittler et al., 2004). The non‐enzymatic antioxidant system consists of various reducing substances such as ascorbic acid (AsA), vitamin E, cytochrome f, and anthocyanins (Noctor, 2005).

We also analyzed the expression level and GO enrichment of genes in the *fls2* mutant, *rbohd/f* double mutant, and WT samples that were related to antioxidant defense metabolites (Figure S6a,b). The level of many genes related to antioxidant defense in *rbohd/f* double mutant samples was mostly higher than they were in WT under both stress and CK conditions. For example, the level of expression of *GLUTATHIONE S‐TRANSFERASE F3* (*GSTF3*) and *MONODEHYDROASCORBATE REDUCTASE 1* (*MDAR1*) was higher in *rbohd/f*_Drought and *rbohd/f*_NaCl samples than in WT_Drought and WT_NaCl samples (Figure S6a). Interestingly, the level of expression of genes related to antioxidative stress in the *fls2* mutant did exhibit significant differences from the WT under either NaCl or drought conditions (Figure S6a).

### 3.7 Fls2‐ RBOHD module co‐regulates the expression of key metabolites

**FIGURE 7:**
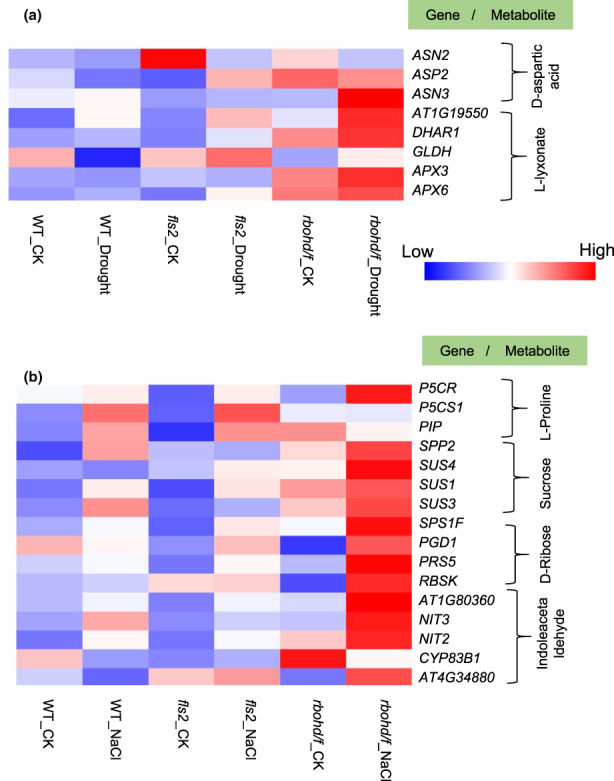
Analysis of the expression of genes related to common differentially abundant metabolites in WT, *fls2* mutant, and *rbohd/f* double mutants under drought and salt stress conditions. (a) Heat map of metabolite‐related gene expression in *fls2*_Drought/CK, *rbohd/f*_Drought/CK, and WT_Drought/CK samples. (b) Heat map of metabolite‐related gene expression in *fls2*_NaCl/CK, *rbohd/f*_NaCl/CK, and WT_NaCl/CK samples.

We further analyzed the expression of identical metabolites and related synthetic genes produced in WT, *fls2*, and *rbohd/f* mutants under drought and salt stress. The results showed that DAA and L‐lyxonate were accumulated in WT, *fls2*, and *rbohd/f* double mutants under drought conditions. The expressions of *ASP2*, *DEHYDROASCORBATE REDUCTASE 1* (*DHAR1*), and *ASCORBATE PEROXIDASE 3* (*APX3*) involved in the synthesis of DAA and L‐lyxonate were also increased correspondingly (Figure 7a). Under salt stress, the contents of L‐proline, sucrose, D‐ribose, and indolealdehyde were increased in WT, *fls2*, and *rbohd/f* double mutants, and the expressions of related genes, such as *P5CR*, *sucrose synthetase 4* (*SUS4*), *AT4G34880*, and *6‐phosphogluconate dehydrogenase 1* (*PGD1*), were also increased (Figure 7b).

## 4 DISCUSSION

### 4.1 FLS2 regulates the response of plants to drought and salt stress by modulating metabolite levels in plants

Plants are inevitably affected by abiotic stresses such as drought, salinity, or high and low temperatures, which greatly limits their growth and development (Nadarajah, 2020). FLS2 is a kinase that functions as a receptor of flg22, a conserved 22 amino acid peptide of plant–bacterial pathogens (Zipfel et al., 2004). Our previous study revealed the important role that FLS2 also plays in regulating the response of plants to abiotic stresses (Liu et al., 2022). In the present study, we further examined the potential effect of FLS2 on the accumulation of metabolites in plants subjected to salt and drought stress. Our results revealed an increased abundance of amino acids and carbohydrates, such as L‐proline, L‐isoleucine, D‐ribose, and others in the “*fls2*_NaCl versus WT_NaCl” comparison group (Figure 2a,b). L‐proline contributes to maintaining the homeostasis of the intracellular redox environment and intracellular ROS content, both of which have been shown to be associated with abiotic stress tolerance (Krishnan et al., 2008; Szabados & Savouré, 2010). Carbohydrates are important carbon energy reserves and are also involved in intracellular redox homeostasis (Poltronieri et al., 2011). The substantial accumulation of metabolites in *fls2*_NaCl mutant plants is due to the sensitivity of *fls2* mutant to salt stress. FLS2 is a receptor kinase, which suggests that it may be involved in the perception of salt stress, a premise indicated in our previous study (Liu et al., 2022). The sensitivity of *fls2* mutant to salt stress may be due to the defective receptor kinases in *fls2* mutant that sense abiotic stress. Notably, in the analysis of the “*fls2*_Drought versus *fls2*_CK” comparison group, no significant accumulation of amino acids or carbohydrates was observed in *fls2*_Drought samples (Figure 6a,b). Based on these data, we speculate that this may indicate that *fls2* mutant does not have a strong sensitivity to drought stress but rather are likely to be tolerant to drought conditions.

### 4.2 *rbohd*/*f* double mutant exhibits hypersensitive changes in the level of metabolites under drought and salt stress conditions

RESPIRATORY BURST OXIDASE HOMOLOG D functions as an essential regulator of ROS production in plants. In plants, the activation of RBOHD activity in response to salt stress results in the accumulation of ROS; thus, RBOHD participates in plant response to salt stress (Liu et al., 2022; Luo et al., 2021). The abundance of metabolites in the *rbohd*/*f* double mutant exhibited a high level of sensitivity to salt stress conditions and LC–MS analysis revealed that the abundance of DAMs such as L‐proline, sucrose, epicatechin, indoleacetic acid, and others, increased in *rbohd/f*_NaCl samples, relative to *rbohd/f*_CK samples (Figure 4a). Indoleacetic acid has been reported to regulate root development in response to many abiotic stresses, including salt stress (Korver et al., 2018). Our results indicated that the abundance of DAMs such as dAMP and UDP‐D‐galactose and others decreased in *rbohd/f*_NaCl samples, relative to WT_NaCl samples (Figure 4a,b). In this regard, ROS accumulation has been reported to have a negative impact on sugar and base moieties and results in oxidative damage to DNA (Boiteux et al., 2017). Consequently, this may lead to a decrease in the abundance of metabolites such as dAMP under salt stress conditions. Our results suggest that ROS signaling mediated by RBOHD/F induces dynamic changes in metabolite production in plants under salt stress.

In the analysis of the “*rbohd/f*_Drought versus *rbohd/f*_CK” and “WT_Drought versus WT_CK” comparison groups, the abundance of DAMs such as indoleacetic acid, L‐glutamic acid, L‐aspartic acid, sucrose, and others was significantly greater in *rbohd/f_*Drought samples than in the other samples (Figures S4a and S5a,b). Extensive amino acid accumulation has often been observed in plants growing under abiotic stress conditions, including maize, cotton, *Arabidopsis thaliana*, and others (Huang & Jander, 2017; Ranieri et al., 1989; Showler, 2002). Additionally, the accumulation of indoleacetic acid is known to have a significant effect on plant growth and stress tolerance (Yemelyanov et al., 2020). In the analysis of the “*rbohd/f_*CK versus WT_CK” comparison group, the number of carbohydrates that decreased in abundance was high (Figure 4b), which may indicate that the absence of RBOHD/F under CK conditions results in a decrease in the abundance of metabolites related to synthetic energy. The changes in the number of DAMs in *rbohd/f_*Drought samples under drought stress conditions indicate that the *rbohd/f* double mutant has an increased sensitivity to oxidative stress response, relative to *rbohd/f*_CK samples. These initial results indicate that the potential mechanism by which FLS2 and RBOHD participate in plant response to drought and salt stress is by regulating changes in the abundance of metabolites.

### 4.3 Combined metabolome and transcriptome analysis of the effect of FLS2 and RBOHD on regulating the abundance of stress‐induced metabolites and the expression of associated genes

We conducted a combined metabolome and transcriptome analysis to examine the relationship between DAMs and the expression of their related genes in *fls2* and *rbohd/f* double mutants under drought and NaCl stress conditions (Figures 2c, 4c, and 6c; Figure S5c). The analysis revealed that many genes, including *GSTF2*, *ASN2*, *YUCCA 8* (*YUC8*), *GLUCOSIDE GLUCOHYDROLASE 2* (*TGG2*), and others, that were related to the identified DAMs were significantly upregulated in *fls2*_NaCl samples, relative to WT_NaCl samples, suggesting that the *fls2* mutant was more sensitive to NaCl than WT plants (Figure 2c). Glutathione S‐transferase has been reported plays an important role in maintaining redox homeostasis and reducing oxidative damage (Chen et al., 2012), and GSTF2 is also closely related to the regulation of oxidative stress response in *Arabidopsis* (Lee et al., 2014). ASN2 has been shown to play an important role in the response of *Arabidopsis* to salt stress (Maaroufi‐Dguimi et al., 2011). TGG2 can decrease the accumulation of ROS in *Arabidopsis* through its antioxidant properties (Zhao et al., 2015). The upregulation of these genes, however, may indicate the increased sensitivity of *fls2* mutant to NaCl (Figure 2c). Although maltose‐related genes exhibited increased expression in *fls2*_Drought samples (Figure 6c), other genes such as *GSTF7* were also highly expressed in WT_CK samples, and thus, did not exhibit a significant increase in *fls2*_Drought samples (Figure 6b). This is consistent with the metabolome data for *fls2* mutant indicating that they exhibited tolerance to drought stress (Figure 6b).

The expression level of genes such as *PYRROLINE‐5‐CARBOXYLATE REDUCTASE* (*P5CR*), *flavonol synthase 1* (*FLS1*), *NIT2*, and others that are related to the synthesis of L‐proline, epicatechin, and indoleacetic acid was higher in *rbohd/f*_NaCl and *rbohd/f*_Drought samples than in WT_NaCl and WT_Drought samples (Figure 4c; Figure S5c). These results are consistent with the metabolome data (Figure 4a; Figure S5a). P5CR is a key enzyme involved in proline biosynthesis and is associated with enhanced drought tolerance in *Arabidopsis* (Chen et al., 2021). FLS2 also plays a key role in plant tolerance to abiotic stress by controlling flavonol accumulation (Zhang et al., 2020). Our results suggest that the *rbohd/f* double mutant is more sensitive to drought and salt stress, and that both RBOHD and FLS2 are required for plant response and adaptation to abiotic stress.

The expression of genes related to antioxidant defense, such as *CATALASE 2* (*CAT2*), *GLUTATHIONE PEROXIDASE 1* (*GPX1*), *CAROTENOID CLEAVAGE DIOXYGENASE 7* (*CCD7*), and others, was greater in *rbohd/f* double mutant than in WT plants (Figure S6). CAT2 has been reported to play an important role in ROS scavenging in plants under abiotic stress conditions (Ono et al., 2021) and GPX1 is known to be involved in the detoxification of H_2_O_2_ (Avsian‐Kretchmer et al., 2004). These genes, however, did not exhibit a specific increase in expression in the *fls2* mutant (Figure S6). These data indicate that both RBOHD and FLS2 are essential for regulating gene expression related to antioxidant defense under drought and salt stress conditions.

The present study identified common metabolites and genes regulated by FLS2 and RBOHD under drought and salt stress conditions (Figure 7). DAA and L‐lyxonate accumulated in both *fls2* mutant and *rbohd/f* double mutant under drought conditions, and L‐proline, sucrose, D‐ribose, and indoleacetaldehyde increased in both *fls2* and *rbohd/f* double mutants under salt conditions, and the expression of their related genes, including *P5CR*, *SUS4*, *AT4G34880*, *PGD1*, and others, also increased (Figure 7b). Notably, SUS4 has been reported to be strongly upregulated in wheat in response to drought stress and other abiotic stresses (Wang et al., 2019). Changes in ROS and Ca^2+^ signaling induced by FLS2 and RBOHD result in the production of the same metabolites and enhancement in the expression of their related genes in response to drought and salt stress conditions. The results of our study provide new insights into plant metabolic processes involved in ROS and Ca^2+^ signaling in response to exposure to abiotic stress.

**FIGURE 8:**
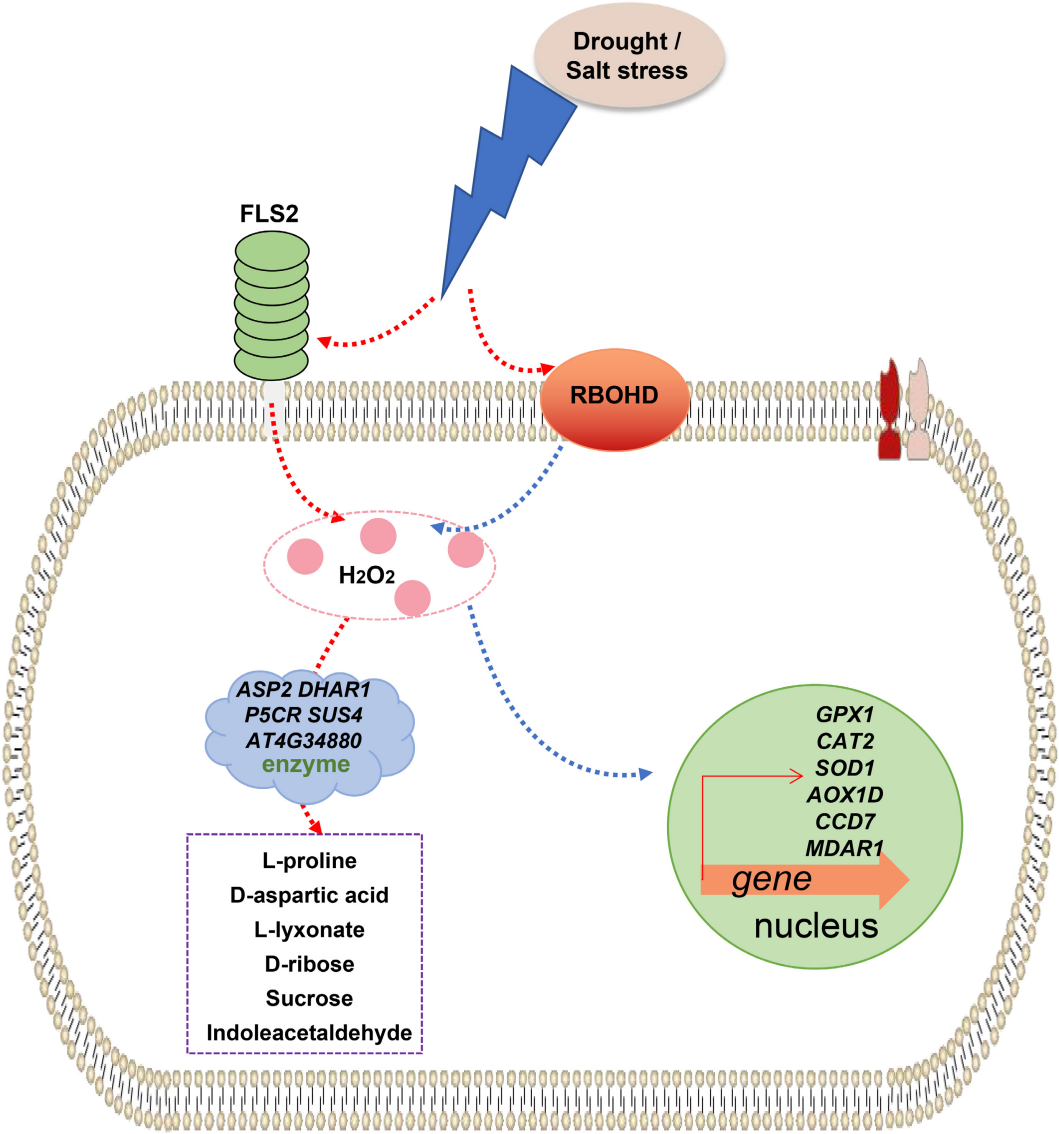
Proposed model of FLAGELLIN SENSITIVE 2 (FLS2) and RESPIRATORY BURST OXIDASE HOMOLOG D (RBOHD) signaling in response to abiotic stress conditions. FLS2 and RBOHD regulate the synthesis of differentially abundant metabolitesthrough reactive oxygen species signaling when plants are subjected to drought and salt stress, which includes the participation of related enzymes. RBOHD also regulates the synthesis of antioxidant enzymes in plants and the expression of their related genes, enabling plants to respond to abiotic stresses.

Both biotic and abiotic stresses can lead to the accumulation of intracellular ROS. Thus, plants respond to both types of stresses by regulating changes in intracellular ROS (Fichman & Mittler, 2020). FLS2 and RBOHD are closely related to the ability of RBOHD to induce ROS and Ca^2+^ signaling, which play an essential role in regulating plant response to abiotic stresses (Noirot et al., 2014). In conclusion, our study revealed the potential role of FLS2 and RBOHD in regulating the abundance of DAMs in response to drought and salt stress conditions and the expression of DAM‐related genes. We provide evidence that the metabolites and genes controlled by both FLS2 and RBOHD regulate ROS and Ca^2+^ signaling in plants subjected to abiotic stress. Our results reveal the potential role of FLS2 and RBOHD in the regulation of the response of a higher plants to abiotic stresses and also provide new strategies for the combined analysis of metabolomic and transcriptomic data (Figure 8).

## CONFLICT OF INTEREST STATEMENT

The authors declare no conflict of interest.

## Supporting information


Figure S1.

Table S1.

## Data Availability

All data supporting the findings of this study are available within the paper and within its supplementary data published online.

## References

[pei310101-bib-0001] Abedi, T. , Khalil, M. F. M. , Koike, K. , Hagura, Y. , Tazoe, Y. , Ishida, N. , Kitamura, K. , & Tanaka, N. (2018). Expression of the human UDP‐galactose transporter gene *hUGT1* in tobacco plants' enhanced plant hardness. Journal of Bioscience and Bioengineering, 126(2), 241–248. 10.1016/j.jbiosc.2018.03.002 29650365

[pei310101-bib-0002] Acosta‐Motos, J. R. , Ortuo, M. F. , Bernal‐Vicente, A. , Diaz‐Vivancos, P. , & Hernandez, J. A. (2017). Plant responses to salt stress: Adaptive mechanisms. Agronomy, 7(1), 18.

[pei310101-bib-0003] Amarowicz, R. , & Weidner, S. (2009). Biological activity of grapevine phenolic compounds. In K. A. Roubelakis‐Angelakis (Ed.), Grapevine molecular physiology & biotechnology (pp. 389–405). Springer Netherlands.

[pei310101-bib-0004] Arif, Y. , Singh, P. , Siddiqui, H. , Bajguz, A. , & Hayat, S. (2020). Salinity induced physiological and biochemical changes in plants: An omic approach towards salt stress tolerance. Plant Physiology and Biochemistry, 156, 64–77. 10.1016/j.plaphy.2020.08.042 32906023

[pei310101-bib-0005] Avsian‐Kretchmer, O. , Gueta‐Dahan, Y. , Lev‐Yadun, S. , Gollop, R. , & Ben‐Hayyim, G. (2004). The salt‐stress signal transduction pathway that activates the *gpx1* promoter is mediated by intracellular H_2_O_2_, different from the pathway induced by extracellular H_2_O_2_ . Plant Physiology, 135(3), 1685–1696. 10.1104/pp.104.041921 15247370 PMC519082

[pei310101-bib-0006] Boiteux, S. , Coste, F. , & Castaing, B. (2017). Repair of 8‐oxo‐7,8‐dihydroguanine in prokaryotic and eukaryotic cells: Properties and biological roles of the Fpg and OGG1 DNA N‐glycosylases. Free Radical Biology and Medicine, 107, 179–201. 10.1016/j.freeradbiomed.2016.11.042 27903453

[pei310101-bib-0007] Bush, D. S. (1995). Calcium regulation in plant cells and its role in signaling. Annual Review of Plant Physiology and Plant Molecular Biology, 46(1), 95–122. 10.1146/annurev.pp.46.060195.000523

[pei310101-bib-0008] Chen, C. , Cui, X. , Zhang, P. , Wang, Z. , & Zhang, J. (2021). Expression of the *pyrroline‐5‐carboxylate reductase* (*P5CR*) gene from the wild grapevine *Vitis yeshanensis* promotes drought resistance in transgenic *Arabidopsis* . Plant Physiology and Biochemistry, 168, 188–201. 10.1016/j.plaphy.2021.10.004 34649022

[pei310101-bib-0009] Chen, J. H. , Jiang, H. W. , Hsieh, E. J. , Chen, H. Y. , Chien, C. T. , Hsieh, H. L. , & Lin, T. P. (2012). Drought and salt stress tolerance of an Arabidopsis glutathione S‐transferase U17 knockout mutant are attributed to the combined effect of glutathione and abscisic acid. Plant Physiology, 158(1), 340–351. 10.1104/pp.111.181875 22095046 PMC3252094

[pei310101-bib-0010] Chi, Y. , Wang, C. , Wang, M. , Wan, D. , Huang, F. , Jiang, Z. , Crawford, B. M. , Vo‐Dinh, T. , Yuan, F. , Wu, F. , & Pei, Z. M. (2021). Flg22‐induced Ca^2+^ increases undergo desensitization and resensitization. Plant Cell & Environment, 44(12), 3563–3575. 10.1111/pce.14186 34536020

[pei310101-bib-0011] Chinchilla, D. , Bauer, Z. , Regenass, M. , Boller, T. , & Felix, G. (2006). The *Arabidopsis* receptor kinase FLS2 binds flg22 and determines the specificity of flagellin perception. Plant Cell, 18(2), 465–476. 10.1105/tpc.105.036574 16377758 PMC1356552

[pei310101-bib-0012] Chinchilla, D. , Zipfel, C. , Robatzek, S. , Kemmerling, B. , Nürnberger, T. , Jones, J. D. , Felix, G. , & Boller, T. (2007). A flagellin‐induced complex of the receptor FLS2 and BAK1 initiates plant defence. Nature, 448(7152), 497–500. 10.1038/nature05999 17625569

[pei310101-bib-0013] Fàbregas, N. , & Fernie, A. R. (2019). The metabolic response to drought. Journal of Experimental Botany, 70(4), 1077–1085. 10.1093/jxb/ery437 30726961

[pei310101-bib-0014] Fichman, Y. , & Mittler, R. (2020). Rapid systemic signaling during abiotic and biotic stresses: Is the ROS wave master of all trades? Plant Journal, 102(5), 887–896. 10.1111/tpj.14685 31943489

[pei310101-bib-0015] Gill, S. S. , & Tuteja, N. (2010). Reactive oxygen species and antioxidant machinery in abiotic stress tolerance in crop plants. Plant Physiology and Biochemistry, 48(12), 909–930. 10.1016/j.plaphy.2010.08.016 20870416

[pei310101-bib-0016] Gómez‐Gómez, L. , & Boller, T. (2000). FLS2: An LRR receptor‐like kinase involved in the perception of the bacterial elicitor flagellin in *Arabidopsis* . Molecular Cell, 5(6), 1003–1011. 10.1016/s1097-2765(00)80265-8 10911994

[pei310101-bib-0017] Huang, T. , & Jander, G. (2017). Abscisic acid‐regulated protein degradation causes osmotic stress‐induced accumulation of branched‐chain amino acids in *Arabidopsis thaliana* . Planta, 246(4), 737–747. 10.1007/s00425-017-2727-3 28668976

[pei310101-bib-0018] Isah, T. (2019). Stress and defense responses in plant secondary metabolites production. Biological Research, 52(1), 39. 10.1186/s40659-019-0246-3 31358053 PMC6661828

[pei310101-bib-0019] Jolliffe, I. T. , & Cadima, J. (2016). Principal component analysis: A review and recent developments. Philosophical Transactions: Mathematical Physical and Engineering Sciences, 374(2065), 20150202. 10.1098/rsta.2015.0202 PMC4792409 26953178

[pei310101-bib-0020] Khan, N. , Bano, A. , Rahman, M. A. , Guo, J. , Kang, Z. , & Babar, M. A. (2019). Comparative physiological and metabolic analysis reveals a complex mechanism involved in drought tolerance in chickpea (*Cicer arietinum* L.) induced by PGPR and PGRs. Scientific Reports, 9(1), 2097. 10.1038/s41598-019-38702-8 30765803 PMC6376124

[pei310101-bib-0021] Korver, R. A. , Koevoets, I. T. , & Testerink, C. (2018). Out of shape during stress: A key role for auxin. Trends in Plant Science, 23(9), 783–793. 10.1016/j.tplants.2018.05.011 29914722 PMC6121082

[pei310101-bib-0022] Krishnan, N. , Dickman, M. B. , & Becker, D. F. (2008). Proline modulates the intracellular redox environment and protects mammalian cells against oxidative stress. Free Radical Biology and Medicine, 44(4), 671–681. 10.1016/j.freeradbiomed.2007.10.054 18036351 PMC2268104

[pei310101-bib-0023] Kumar, M. , Kumar Patel, M. , Kumar, N. , Bajpai, A. B. , & Siddique, K. H. M. (2021). Metabolomics and molecular approaches reveal drought stress tolerance in plants. International Journal of Molecular Sciences, 22(17). 10.3390/ijms22179108 PMC8431676 34502020

[pei310101-bib-0024] Lee, S. H. , Li, C. W. , Koh, K. W. , Chuang, H. Y. , Chen, Y. R. , Lin, C. S. , & Chan, M. T. (2014). MSRB7 reverses oxidation of GSTF2/3 to confer tolerance of *Arabidopsis thaliana* to oxidative stress. Journal of Experimental Botany, 65(17), 5049–5062. 10.1093/jxb/eru270 24962998 PMC4144780

[pei310101-bib-0025] Li, L. , Li, M. , Yu, L. , Zhou, Z. , Liang, X. , Liu, Z. , Cai, G. , Gao, L. , Zhang, X. , Wang, Y. , Chen, S. , & Zhou, J.‐M. (2014). The FLS2‐associated kinase BIK1 directly phosphorylates the nadph oxidase rbohd to control plant immunity. Cell Host & Microbe, 15(3), 329–338. 10.1016/j.chom.2014.02.009 24629339

[pei310101-bib-0026] Liu, Z. , Guo, C. , Wu, R. , Hu, Y. , Zhou, Y. , Wang, J. , Yu, X. , Zhang, Y. , Bawa, G. , & Sun, X. (2022). FLS2‐RBOHD‐PIF4 module regulates plant response to drought and salt stress. International Journal of Molecular Sciences, 23(3). 10.3390/ijms23031080 PMC8835674 35163000

[pei310101-bib-0027] Luo, X. , Dai, Y. , Zheng, C. , Yang, Y. , Chen, W. , Wang, Q. , Chandrasekaran, U. , du, J. , Liu, W. , & Shu, K. (2021). The ABI4‐RbohD/VTC2 regulatory module promotes reactive oxygen species (ROS) accumulation to decrease seed germination under salinity stress. New Phytologist, 229(2), 950–962. 10.1111/nph.16921 32916762

[pei310101-bib-0028] Maaroufi‐Dguimi, H. , Debouba, M. , Gaufichon, L. , Clément, G. , Gouia, H. , Hajjaji, A. , & Suzuki, A. (2011). An *Arabidopsis* mutant disrupted in *ASN2* encoding asparagine synthetase 2 exhibits low salt stress tolerance. Plant Physiology and Biochemistry, 49(6), 623–628. 10.1016/j.plaphy.2011.03.010 21478030

[pei310101-bib-0029] Majumdar, R. , Barchi, B. , Turlapati, S. A. , Gagne, M. , Minocha, R. , Long, S. , & Minocha, S. C. (2016). Glutamate, ornithine, arginine, proline, and polyamine metabolic interactions: The pathway is regulated at the post‐transcriptional level. Frontiers in Plant Science, 7, 78. 10.3389/fpls.2016.00078 26909083 PMC4754450

[pei310101-bib-0030] Melotto, M. , Underwood, W. , Koczan, J. , Nomura, K. , & He, S. Y. (2006). Plant stomata function in innate immunity against bacterial invasion. Cell, 126(5), 969–980. 10.1016/j.cell.2006.06.054 16959575

[pei310101-bib-0031] Mittler, R. , Vanderauwera, S. , Gollery, M. , & Breusegem, F. V. (2004). Reactive oxygen gene network of plants. Trends in Plant Science, 9(10), 490–498. 15465684 10.1016/j.tplants.2004.08.009

[pei310101-bib-0032] Nadarajah, K. K. (2020). ROS homeostasis in abiotic stress tolerance in plants. International Journal of Molecular Sciences, 21(15). 10.3390/ijms21155208 PMC7432042 32717820

[pei310101-bib-0033] Nicholson, J. K. , Lindon, J. C. , & Holmes, E. (1999). 'Metabonomics': Understanding the metabolic responses of living systems to pathophysiological stimuli via multivariate statistical analysis of biological NMR spectroscopic data. Xenobiotica, 29(11), 1181–1189. 10.1080/004982599238047 10598751

[pei310101-bib-0034] Noctor, F. G. (2005). Redox homeostasis and antioxidant signaling: A metabolic interface between stress perception and physiological responses. Plant Cell, 17(7), 1866–1875. 15987996 10.1105/tpc.105.033589 PMC1167537

[pei310101-bib-0035] Noctor, G. , Mhamdi, A. , & Foyer, C. H. (2014). The roles of reactive oxygen metabolism in drought: Not so cut and dried. Plant Physiology, 164(4), 1636–1648. 24715539 10.1104/pp.113.233478 PMC3982730

[pei310101-bib-0036] Noirot, E. , Der, C. , Lherminier, J. , Robert, F. , Moricova, P. , Kiêu, K. , Leborgne‐Castel, N. , Simon‐Plas, F. , & Bouhidel, K. (2014). Dynamic changes in the subcellular distribution of the tobacco ROS‐producing enzyme RBOHD in response to the oomycete elicitor cryptogein. Journal of Experimental Botany, 65(17), 5011–5022. 10.1093/jxb/eru265 24987013 PMC4144778

[pei310101-bib-0037] Okada, T. , Afendi, F. M. , Altaf‐Ul‐Amin, M. , Takahashi, H. , Nakamura, K. , & Kanaya, S. (2010). Metabolomics of medicinal plants: The importance of multivariate analysis of analytical chemistry data. Current Computer Aided Drug Design, 6(3), 179–196. 10.2174/157340910791760055 20550511

[pei310101-bib-0038] Ono, M. , Isono, K. , Sakata, Y. , & Taji, T. (2021). CATALASE2 plays a crucial role in long‐term heat tolerance of *Arabidopsis thaliana* . Biochemical and Biophysical Research Communications, 534, 747–751. 10.1016/j.bbrc.2020.11.006 33199020

[pei310101-bib-0039] Pang, Z. , Zhou, G. , Ewald, J. , Chang, L. , Hacariz, O. , Basu, N. , & Xia, J. (2022). Using MetaboAnalyst 5.0 for LC–HRMS spectra processing, multi‐omics integration and covariate adjustment of global metabolomics data. Nature Protocols., 17, 1735–1761. 10.1038/s41596-022-00710-w 35715522

[pei310101-bib-0040] Poltronieri, P. , Bonsegna, S. , De, D. S. , & Santino, A. (2011). Molecular mechanisms in plant abiotic stress response. Ratarstvo I Povrtarstvo, 48(1), 15–24.

[pei310101-bib-0041] Ranieri, A. , Bernardi, R. , Lanese, P. , & Soldatini, G. F. (1989). Changes in free amino acid content and protein pattern of maize seedlings under water stress. Environmental and Experimental Botany, 29(3), 351–357. 10.1016/0098-8472(89)90009-9

[pei310101-bib-0042] Rhodes, D. , & Samaras, Y. (1994). Genetic control of osmoregulation in plants. In K. Strange (Ed.), Cellular and molecular physiology of cell volume regulation (pp. 347–361). CRC Press.

[pei310101-bib-0043] Showler, A. T. (2002). Effects of water deficit stress, shade, weed competition, and kaolin particle film on selected foliar free amino acid accumulations in cotton, *Gossypium hirsutum* (L.). Journal of Chemical Ecology, 28(3), 631–651. 10.1023/a:1014556515489 11944838

[pei310101-bib-0044] Singh, A. K. , Dhanapal, S. , & Yadav, B. S. (2020). The dynamic responses of plant physiology and metabolism during environmental stress progression. Molecular Biology Reports, 47(2), 1459–1470. 10.1007/s11033-019-05198-4 31823123

[pei310101-bib-0045] Szabados, L. , & Savouré, A. (2010). Proline: a multifunctional amino acid. Trends in Plant Science, 15(2), 89–97. 10.1016/j.tplants.2009.11.009 20036181

[pei310101-bib-0046] Trygg, J. , & Wold, S. (2002). Orthogonal projections to latent structures (O‐PLS). Journal of Chemometrics, 16(3), 119–128. 10.1002/cem.695

[pei310101-bib-0047] Wang, Y. , Zhang, X. , Huang, G. , Feng, F. , Liu, X. , Guo, R. , Gu, F. , Zhong, X. , & Mei, X. (2019). iTRAQ‐based quantitative analysis of responsive proteins under peg‐induced drought stress in wheat leaves. International Journal of Molecular Sciences, 20(11), 2621. 10.3390/ijms20112621 31141975 PMC6600531

[pei310101-bib-0048] Xie, Y. J. , Xu, S. , Han, B. , Wu, M. Z. , & Shen, W. B. (2011). Evidence of *Arabidopsis* salt acclimation induced by up‐regulation of *HY1* and the regulatory role of RbohD‐derived reactive oxygen species synthesis. Plant Journal, 66(2), 280–292. 10.1111/j.1365-313X.2011.04488.x 21205037

[pei310101-bib-0049] Yang, L. , Wen, K. S. , Ruan, X. , Zhao, Y. X. , Wei, F. , & Wang, Q. (2018). Response of plant secondary metabolites to environmental factors. Molecules, 23(4). 10.3390/molecules23040762 PMC6017249 29584636

[pei310101-bib-0050] Yang, L. L. , Yang, L. , Yang, X. , Zhang, T. , Lan, Y. M. , Zhao, Y. , Han, M. , & Yang, L. M. (2020). Drought stress induces biosynthesis of flavonoids in leaves and saikosaponins in roots of *Bupleurum chinense* DC. Phytochemistry, 177, 112434. 10.1016/j.phytochem.2020.112434 32544729

[pei310101-bib-0051] Yemelyanov, V. V. , Lastochkin, V. V. , Chirkova, T. V. , Lindberg, S. M. , & Shishova, M. F. (2020). Indoleacetic acid levels in wheat and rice seedlings under oxygen deficiency and subsequent reoxygenation. Biomolecules, 10(2). 10.3390/biom10020276 PMC7072260 32054127

[pei310101-bib-0052] Yu, C. W. , Murphy, T. M. , Sung, W. W. , & Lin, C. H. (2002). H_2_O_2_ treatment induces glutathione accumulation and chilling tolerance in mung bean. Functional Plant Biology, 29, 1081–1087. 32689559 10.1071/PP01264

[pei310101-bib-0053] Zhang, X. , Yang, H. , Schaufelberger, M. , Li, X. , Cao, Q. , Xiao, H. , & Ren, Z. (2020). Role of flavonol synthesized by nucleus fls1 in *Arabidopsis* resistance to pb stress. Journal of Agricultural and Food Chemistry, 68(36), 9646–9653. 10.1021/acs.jafc.0c02848 32786845

[pei310101-bib-0054] Zhao, Y. , Wang, J. , Liu, Y. , Miao, H. , Cai, C. , Shao, Z. , Guo, R. , Sun, B. , Jia, C. , Zhang, L. , Gigolashvili, T. , & Wang, Q. (2015). Classic myrosinase‐dependent degradation of indole glucosinolate attenuates fumonisin B1‐induced programmed cell death in *Arabidopsis* . Plant Journal, 81(6), 920–933. 10.1111/tpj.12778 25645692

[pei310101-bib-0055] Zhou, Y. , Zhou, B. , Pache, L. , Chang, M. , Khodabakhshi, A. H. , Tanaseichuk, O. , Benner, C. , & Chanda, S. K. (2019). Metascape provides a biologist‐oriented resource for the analysis of systems‐level datasets. Nature Communications, 10(1), 1523. 10.1038/s41467-019-09234-6 PMC6447622 30944313

[pei310101-bib-0056] Zipfel, C. , Robatzek, S. , Navarro, L. , Oakeley, E. J. , Jones, J. D. , Felix, G. , & Boller, T. (2004). Bacterial disease resistance in *Arabidopsis* through flagellin perception. Nature, 428(6984), 764–767. 10.1038/nature02485 15085136

