# Supplementary material for: FLS2‐RBOHD module regulates changes in the metabolome of Arabidopsis in response to abiotic stress
Source: Plant Environ Interact. 2023 Feb 9;4(1):36–54. doi: 10.1002/pei3.10101 (PMC10168046; doi:10.1002/pei3.10101)
Supplement: Supplementary file 1 — Figure S1. [file PEI3-4-36-s001.docx]

**Supporting information**

The following supplemental materials are available.

Figure S1. LC-MS identification of differential metabolites.

Figure S2. PCA plot of all samples.

Figure S3. Verification of the T‐DNA insertion of mutants.

Figure S4. Metabolic network diagram of DAMs in ‘*rbohd/f*_Drought vs. WT_Drought’ comparison groups.

Figure S5. Identification and analysis of the DAMs in different comparison groups under control and drought conditions.

Figure S6. Analysis of antioxidant defense-related gene expression in different comparison groups under CK, drought, and salt stress conditions.

Table S1. List of mutant lines.

Table S2. List of oligonucleotides.

Table S3. Differential metabolites in WT-NaCl vs WT-CK.

Table S4. Differential metabolites in *fls2*-NaCl vs *fls2*-CK.

Table S5. Differential metabolites in *rbohd/f*-NaCl vs *rbohd/f*-CK.

Table S6. Differential metabolites in WT-Drought vs WT-CK.

Table S7. Differential metabolites in *fls2*-Drought vs *fls2*-CK.

Table S8. Differential metabolites in *rbohd/f*-Drought vs *rbohd/f*-CK.

Table S9. Differential metabolites in *fls2*-CK vs WT-CK.

Table S10. Differential metabolites in *rbohd/f* -CK vs WT-CK.

Table S11. Differential metabolites in *fls2*-NaCl vs WT-NaCl.

Table S12. Differential metabolites in *rbohd/f* -NaCl vs WT-NaCl.

Table S13. Differential metabolites in *fls2*-Drought vs WT-Drought.

Table S14. Differential metabolites in *rbohd/f*-Drought vs WT-Drought.

Table S15. Screened differential metabolites-associated metabolic enzymes in *fls2*-NaCl vs WT-NaCl.

Table S16. Screened differential metabolites-associated metabolic enzymes in *rbohd/f*-NaCl vs WT-NaCl.

Table S17. Screened differential metabolites-associated metabolic enzymes in *fls2*- Drought vs WT- Drought.

Table S18. Screened differential metabolites-associated metabolic enzymes in *rbohd/f*-Drought vs WT-Drought.

**Figure S1. LC-MS identification of differential metabolites.**

(a) The base peak chromatogram in positive ion mode, of WT, *fls2*, and *rbohd/f* sample ions under CK, NaCl, and drought conditions. (b) Principal component analysis (PCA) of metabolites in the comparison of WT and *fls2*, and *rbohd/f* samples under either salt or drought stress conditions. (c) The orthogonal partial least squares analysis (OPLS-DA) of metabolites in the same comparison as (b).

**Figure S2. PCA plot of all samples.**

PCA analysis of *fls2*-Drought/NaCl/CK, *rbohd/f*-Drought/NaCl/CK, WT-Drought/NaCl/CK, and quality control (QC) samples.

**Figure S3. Verification of the T‐DNA insertion of mutants.** Identification of the homozygotes of *fls2* mutants (SALK_141277)**.** DNA was extracted from the mutants and WT; the location of the T‐DNA insertion in each mutant was verified by PCR with the corresponding primers.

**Figure S4. Metabolic network diagram of DAMs in ‘*rbohd/f*_Drought vs. WT_Drought’ comparison groups.**

(a) Metabolic network diagram of DAMs in the ‘*rbohd/f*_Drought vs. WT_Drought’ comparison group. Solid lines indicate one-step reactions; dashed lines indicate two- or more-step reactions. (b) Impact of DAMs on different metabolic pathways in the ‘*rbohd/f*_Drought vs. WT_Drought’ comparison group. All matching pathways for metabolites were determined based on p-values in a pathway enrichment analysis and pathway impact values were derived from a pathway topology analysis.

**Figure S5. Identification and analysis of the DAMs in different comparison groups under control and drought conditions.**

(a) Volcano plots of DAMs in the various comparisons of *rbohd/f* and WT samples under CK and drought stress conditions. DAMs were identified based on a p-value < 0.05 and |log2FC| > 0.58. Red dots represent DAMs with significantly increased abundance in the comparison groups, green dots represent metabolites with significantly decreased abundance, and the gray dots represent metabolites whose change was non-significant. (b) Bar chart of the number of metabolites in different categories of metabolites in the ‘*rbohd/f*_Drought vs. WT_Drought’ comparison group. (c) Heat map of the expression of metabolite-related genes associated with ‘*rbohd/f*_Drought vs. WT_Drought’. Heat map of the expression of genes related to the synthesis of L-proline, L-glutamic acid, sucrose, indoleacetic acid, AMP, and UDP-D-galactose in *rbohd/f*_Drought, *rbohd/f*_CK, WT_CK, WT_Drought samples are presented. (d) Heat map of gene expression of metabolite synthesis-related genes identified in GO enrichment analysis. Genes presented are related to (c).

**Figure S6.** **Analysis of antioxidant defense-related gene expression in different comparison groups under CK, drought, and salt stress conditions.**

(a) Heat map of the expression level of genes associated with the synthesis of superoxide dismutase, ascorbate peroxidase, catalase, glutathione peroxidase, dehydroascorbate reductase, monodehydroascorbate reductase, thioredoxin peroxidase, alternative oxidase and carotenoid, vitamin E, cytochrome f, and anthocyanin in *fls2*_Drought/NaCl/CK, *rbohd/f*_Drought/NaCl/CK, and WT_Drought/NaCl/CK samples. (b) Heat map of the expression of GO enrichment analysis genes related to the annotated metabolites in (a).
